# Visualizing an Umbilical Venous Catheter in Malposition Using POCUS

**DOI:** 10.24908/pocusj.v11i01.19974

**Published:** 2026-04-22

**Authors:** Jacob Kelner, Douglas Moote

**Affiliations:** 1 Department of Pediatrics, University of Connecticut School of Medicine, Farmington, CT, USA; 2 Division of Neonatology, Connecticut Children's, Hartford, CT, USA; 3 Departments of Pediatrics and Diagnostic Imaging and Therapeutics, University of Connecticut School of Medicine, Farmington, CT, USA; 4 Division of Pediatric Radiology, Connecticut Children's, Hartford, CT, USA

**Keywords:** Neonatal Intensive Care Unit, Neonate, Umbilical Venous Catheter, Malposition, Point-of-Care Ultrasound, POCUS

## Abstract

Umbilical venous catheters (UVCs) are commonly needed in critically ill infants in the neonatal intensive care unit (NICU). However, there is an increased risk for major adverse events with improper positioning. There is a strong argument for using point of care ultrasound (POCUS) to determine catheter tip positioning during and after UVC placement, but X-rays remain the gold standard in many NICUs. This case reports a UVC in malposition in the liver viewed on POCUS after appearing to be in the correct position on both anteroposterior (AP) and lateral X-rays.

## Introduction

Umbilical venous catheters (UVCs) are commonly placed in critically ill newborn infants for stable vascular access [1,2]. During insertion and dwell time, tip migration and malposition can lead to major adverse events if they injure the surrounding vessel, tissue, or organs [2]. Radiography remains the typical standard of care for assessing UVC tip positioning [3,4]. At our institution, correct positioning of a UVC is defined as having the catheter tip at the level of the T9 vertebral body—at or just above the diaphragm—on the anteroposterior (AP) X-ray, or if the catheter tip is posterior to the cardiac silhouette—at or just above the diaphragm—on the lateral view.

Several reports raise concerns about the accuracy of radiography for UVC positioning and have proposed ultrasound as an alternative modality [1,5–8]. A prospective observational study using POCUS to detect UVC migration found that among 40 infants with UVCs, 63% migrated within the first 7 days, and 68% of these migrations were in malposition [9]. Most commonly, they were found within the heart. More concerning was that only 11% of the UVCs in malposition were detected by X-ray.

While a limited number of case reports have used POCUS to diagnose an umbilical line in malposition, none have demonstrated a UVC that appeared correctly positioned on both AP and lateral X-rays prior to POCUS assessment by a neonatal provider, as this case report does [10,11].

## Case Presentation

A term neonate boy was born at an outside hospital via vaginal delivery, complicated by maternal placental abruption and a shoulder dystocia requiring forceps. Apgar scores were 1, 3, and 3 at 1, 5, and 10 minutes of life, respectively. The cord blood gas indicated severe acidosis (pH of 6.70; base deficit of -18). He was intubated in the delivery room, and a low-lying UVC was secured. Our transport team initiated therapeutic hypothermia for concerns of hypoxic-ischemic encephalopathy, and he was transported to our tertiary academic neonatal intensive care unit (NICU) for further care.

**Figure 1.**
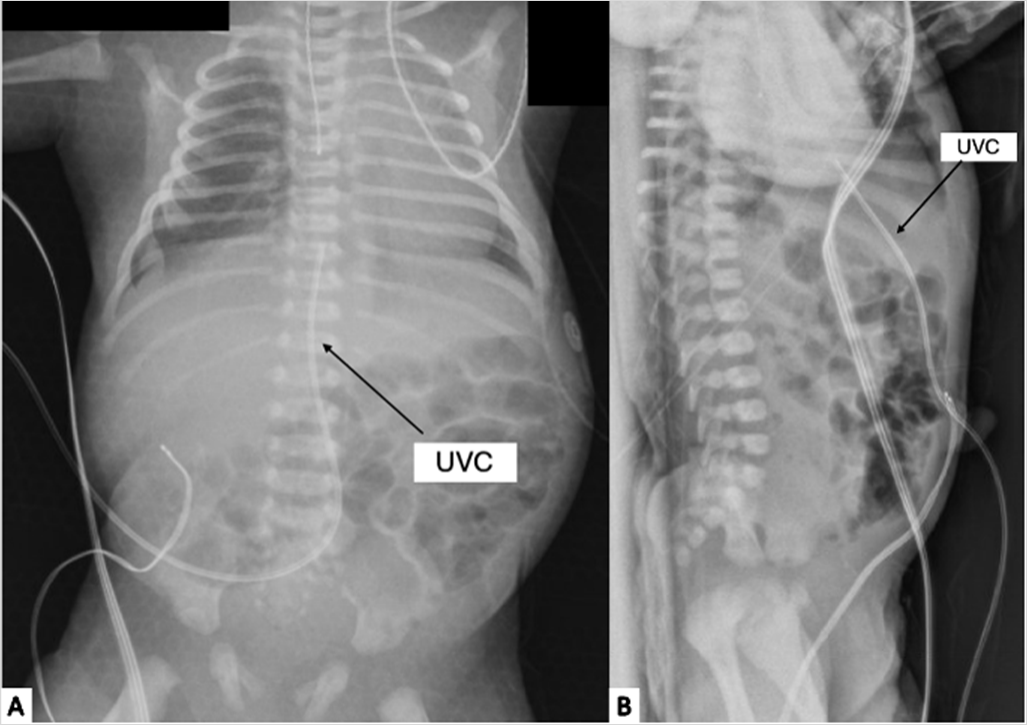
AP (A) and lateral (B) chest/abdomen radiographs with UVC at 10 cm. The UVC appears to be approximately 1 cm from the CAJ. AP, Anteroposterior; CAJ, Cavo-Atrial Junction; UVC, Umbilical Venous Catheter.

**Figure 2.**
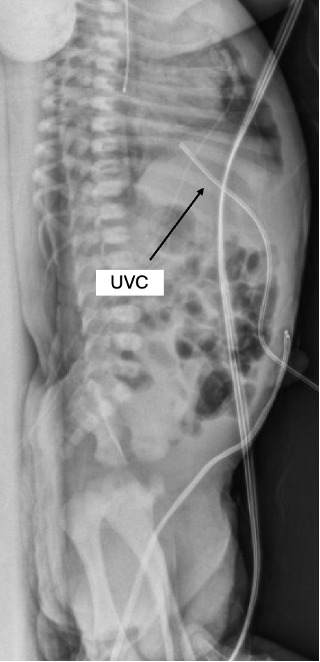
Lateral chest/abdomen radiograph after the UVC advanced to 11 cm. The same radiologist read that the UVC terminates in the region of the inferior CAJ. CAJ, Cavo-Atrial Junction; UVC, Umbilical Venous Catheter.

A UVC was reattempted. Based on his birth weight of 3.72 kg, an estimated best predictive insertion length of 10–11 cm was calculated [12,13]. A 5 Fr umbilical catheter was introduced into the umbilical vein and secured at 10 cm. The catheter flushed well but had no blood return. An AP and lateral chest/abdomen radiograph (Figure 1) showed the UVC to be approximately 1 cm lower than the cavo-atrial junction (CAJ), and the catheter was advanced by 1 cm. The radiologist confirmed the catheter was in the correct position in the CAJ on a repeat lateral radiograph (Figure 2).

**Figure 3.**
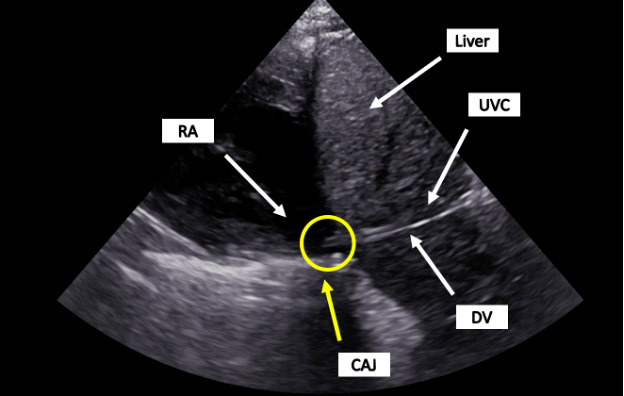
POCUS of a correctly placed UVC. The catheter traverses the DV and ends in the CAJ (circle). CAJ, Cavo-Atrial Junction; DV, Ductus Venosus; POCUS, Point of care ultrasound; RA, Right Atrium; UVC, Umbilical Venous Catheter.

**Figure 4.**
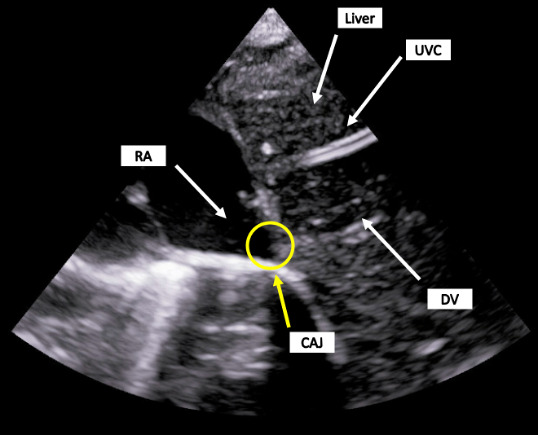
POCUS of the liver and inferior CAJ by the bedside provider. The UVC is not in the DV/CAJ and appears to be within the liver. CAJ, Cavo-Atrial Junction; DV, Ductus Venosus; POCUS, Point of care ultrasound; RA, Right Atrium; UVC, Umbilical Venous Catheter.

**Figure 5.**
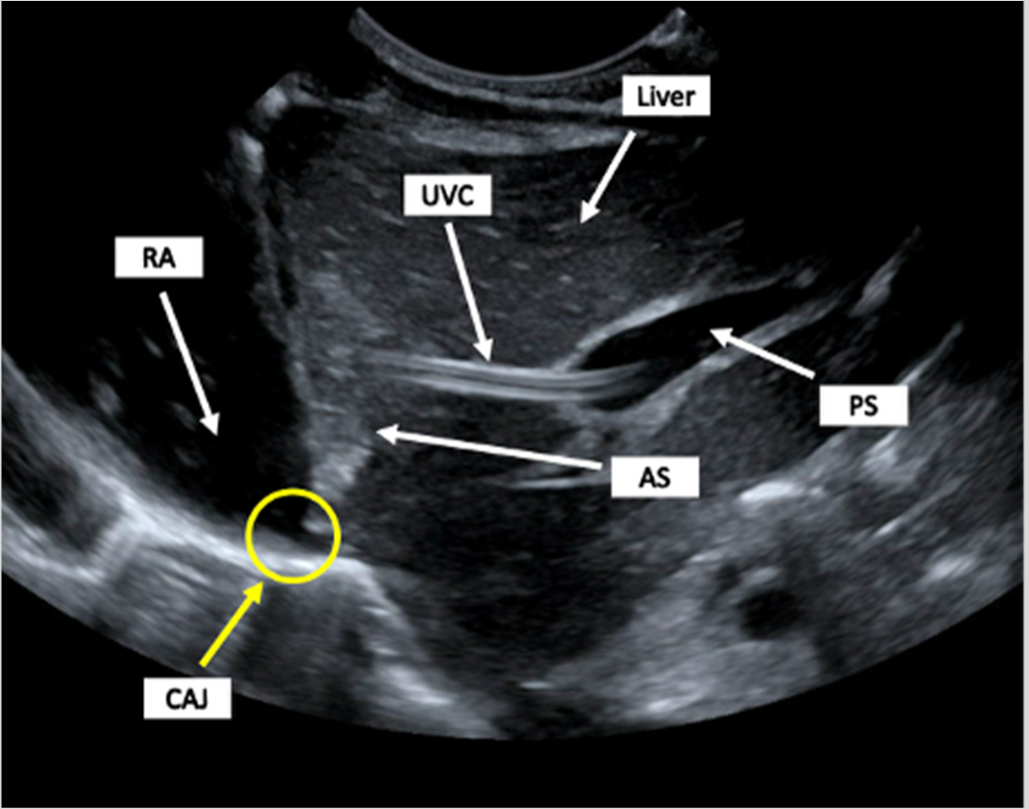
Ultrasound imaging of the liver and CAJ by the radiology department. The radiologist read, *the UVC line is seen extending into the portal vein and then continues into the liver parenchyma anterior to the junction of the hepatic veins and IVC. Injection of a small volume of fluid into the UVC line in the NICU at the time of the ultrasound confirms that the tip of the catheter is abnormally positioned within the liver parenchyma*. AS, Agitated Saline; CAJ, Cavo-Atrial Junction; IVC, Inferior Vena Cava; NICU, Neonatal Intensive Care Unit; PS, Portal Sinus; RA, Right Atrium; UVC, Umbilical Venous Catheter.

Since there was no blood return, POCUS was performed by a trained neonatal provider (JK) shortly after the radiograph to assess the position of the UVC tip. A 12s phased array probe was placed just below the xiphoid process in the sagittal plane, with the probe marker oriented towards the infant's head. While fanning slightly right (normal UVC positioning in Figure 3), no catheter was found in the ductus venosus (DV), CAJ, or the right atrium (RA), but a catheter was seen within the liver (Figure 4, Supplementary Material S1) [14]. The images were uploaded to the infant's chart and reviewed with the on-call radiologist, who was concerned the catheter might be positioned anteriorly in the liver parenchyma. A stat radiology department ultrasound of the liver demonstrated an abnormally positioned UVC line terminating in the liver parenchyma, anterior to the hepatic vein/IVC junction region (Figure 5, Supplementary Material S2). During the radiology department ultrasound, the bedside nurse quickly flushed a small volume of normal saline (NS) through the catheter to create bubbles, which were easily visualized at the UVC tip under ultrasound. The catheter was withdrawn to a low-lying UVC.

## Discussion and Conclusion

This case report highlights a significant pitfall of radiography for confirming tip positioning while simultaneously promoting the use of POCUS. Here, POCUS outperformed the lateral X-ray, which is often added when UVC tip placement is difficult to evaluate on the AP X-ray. In this case, we used a quick flush of NS to “agitate” the saline and create bubbles at the UVC tip, as this can aid in assessing line placement [15]. However, agitating the saline by flushing 2 mL of saline back and forth between two syringes via a stopcock before flushing has been reported to be safe in neonates and improve visualization compared to NS flush alone [16].

POCUS during UVC placement improves the accuracy of tip positioning, reduces catheter adjustments, and decreases the number of X-rays required during the process [17]. The continued reliance on X-rays is due to the considerable barriers to introducing POCUS into NICUs. A lack of training and resources can be challenging to navigate, compounded by a lack of collaboration with radiology. Previously, 55% of NICUs in the United States reported this as a barrier to adopting POCUS [18]. We want to emphasize that POCUS did not replace radiology department ultrasound; instead, it facilitated faster diagnosis by allowing for a quicker request for radiology department ultrasound.

Fostering a strong relationship between the neonatal POCUS and pediatric radiology teams is vital to improving the quality of neonatal care. Based on our institutional experience, this relationship is built on a mutual understanding of the roles that both POCUS and radiology department ultrasound play in achieving a shared mission of improving patient outcomes. Having standardized protocols for acquiring, interpreting, and documenting POCUS images in patient charts can enable the on-call radiologist to provide quality assurance when needed. Lastly, collaborating on research and quality-improvement projects further improves communication between the two departments and leverages both specialists' expertise to advance the field of neonatal echography.

In conclusion, this case report contributes to the growing body of evidence supporting the use of POCUS over X-rays for more accurate and safer UVC positioning. It also provides evidence for neonatal POCUS programs to foster a more collaborative relationship with their radiology department, relieving a significant barrier to introducing POCUS in the NICU.
